# Evaluation of Fillers for Magnesium Potassium Phosphate Cement (MKPC) for the Encapsulation of Low and Intermediate Level Metallic Radioactive Wastes

**DOI:** 10.3390/ma16020679

**Published:** 2023-01-10

**Authors:** Mikel Dieguez, Ana Isabel Ruiz, Jaime Cuevas, María Cruz Alonso, Inés García-Lodeiro, Raúl Fernández

**Affiliations:** 1 Department of Geology and Geochemistry, Faculty of Sciences, Autonomous University of Madrid, Cantoblanco, 28049 Madrid, Spain; 2 Eduardo Torroja Institute for Construction Sciences (IETcc-CSIC), c/Serrano Galvache 4, 28033 Madrid, Spain

**Keywords:** magnesium phosphate cement, metallic radioactive waste, cement filler

## Abstract

This study investigates the effect of coal fly ash (FA), wollastonite (WO), pumice (PM), and metakaolin (MK) as filler materials in the rheological, mechanical, chemical, and mineralogical properties of a magnesium potassium phosphate cement (MKPC), designed for the encapsulation of low and intermediate level radioactive wastes containing reactive metals. Workability, compression strength, dimensional stability, pH, chemical composition, and mineralogical properties were studied in different pastes and mortars of MKPC with a fixed molar ratio of MgO/KH_2_PO_4_ = 1. No new mineral phases were found with the addition of the fillers, denoting their low chemical impact on the MKPC system. Moreover, all formulations with a water/cement mass ratio of <0.65 presented compressive strengths higher than 30 MPa after 90 days, and pH values lower than 8.5, corresponding to the passivation zone of aluminum corrosion.

## 1. Introduction

Magnesium potassium phosphate cements (MKPC) are receiving increasing interest for their use in a wide range of applications, including bone repair biomaterials [1,2], rapid repair of damaged concrete structures that require a short interruption of services, such as some highway pavement or airport runways [3,4], solidification of urban river dredged sludge [5], immobilization of galvanic wastes [6], stabilization and solidification of metals in a biodegradable matrix [7], 3D printing [8,9,10], and encapsulation of radioactive wastes [11,12,13].

Specifically for the encapsulation of low and intermediate level radioactive wastes containing reactive metals, MKPC aims to replace cements based on Ordinary Portland cement (OPC) systems since the high alkaline nature of the latter can induce corrosion of the metals (e.g., aluminum, beryllium, Magnox, and uranium), leading to the generation of hydrogen gas and pressures accumulated in the container with a risk of explosion [12]. Furthermore, expansion of the wastes can occur, due to the formation of corrosion products [14]. MKPC are considered a possible alternative to OPC due to their lower pH between 4 and 9, corresponding to the passivation zone of aluminum [15]. In addition to their near-neutral pH, MKPC have lower water demand, shorter setting time, and higher early compressive strength than OPC, being viable candidates for the substitution of the OPC in this type of application.

MKPC are based on the mix of dead burnt MgO with soluble acid phosphate salt (mostly KH_2_PO_4_) and water according to reaction 1, plus the addition of solid inert components, known as fillers, to reduce the high temperatures at the initial stage of the acid-base reaction, to enhance rheological properties, such as fluidity and workability, improve water resistance, prolong the setting time, modify the mechanical strength and decrease costs [16,17,18]. A chemical retarder is also added in the formulation of MKPC to delay the acid-base reaction, control the setting time and limit the temperature rise. The most commonly used retarders include boric acid and borax [19,20], but other chemicals (thiosulphates, tripolyphosphates, alginates) are also investigated [21]. The stoichiometric reaction between periclase (MgO), monopotassium phosphate (KH_2_PO_4_) and water produce K-struvite (MgKPO_4_·6H_2_O) as a reaction product:

(1) $$\mathrm{MgO}+{\mathrm{KH}}_{2}{\mathrm{PO}}_{4}+5H_{2}O\to {\mathrm{MgKPO}}_{4}\cdot 6H_{2}O+Q_{\mathrm{heat}}$$

To obtain stable pastes and mortars without any segregation, the MKPC formulations require the addition of a filler or a large excess of MgO against KH_2_PO_4_, exceeding the molar ratio of 1 [22,23,24]. However, an excess of MgO could produce potential expansion in wet environments due to the hydration of the excess of MgO into brucite and increase the pH of the system; therefore, the use of a low-cost filler is much more preferred [24]. Coal fly ash (FA) is the most commonly used inert filler until now, due to its spherical morphology and small size in the MKPC matrix, able to enhance the fluidity in the mixture and the long-term strength of the cement [25]. Nevertheless, the availability of FA is being recently reduced as it is generated as a by-product in coal-based thermal power plants that are being shut down worldwide to reduce carbon emissions and meet climate targets. Therefore, an increasing interest in the study of novel types of fillers is generated for the improvement of properties and quality/cost ratio of MKPC.

A proposed alternative for FA substitution is wollastonite (WO), an inosilicate of calcium with the chemical composition of CaSiO_3_. This inosilicate was evaluated by Xu et al. [23] in MKPC pastes with low water/cement (cement = MgO + KH_2_PO_4_) ratios of 0.25 and MgO/KH_2_PO_4_ molar ratios of 2.7. The authors concluded that the presence of wollastonite and high molar ratios MgO/KH_2_PO_4_ of 2.7 in MKPC suppresses the formation of efflorescence, increases compressive strength, and reduces the pH values in the cement formulation.

Metakaolin (MK) has been also considered a filler material in MKPC systems. Studies found an improvement of mechanical properties as compressive strength with the addition of MK, as this attributed to the formation of highly reinforced microstructures and denser interfaces between the metakaolin particles and hydration products, resulting in a decrease in porosity. In addition, metakaolin particles can provide surface sites for the crystallization of hydration products, inducing the generation of a more homogeneous microstructure [26,27].

Pumice (PM) is a porous volcanic rock mainly composed of amorphous silica with a minor proportion of aluminum oxide and other silicates, such as biotite, quartz, and alkali feldspars, and has been studied as a supplementary cementitious material (SCM) in OPC. It has been reported in literature that pumice powder decreases the workability and compressive strength of concrete but improves its durability [28]. Pumice also increases the setting time and decreases the heat of hydration of cement pastes [29,30]. Pumice has lower pozzolanic activity in the early period than alternative SCMs and yet the compressive strength increases with time, as the hydration further evolves with age producing a much denser microstructure in the cement paste [31]. Nonetheless, there are no studies to date on its performance as filler material in MKPC systems.

Regarding the chemical and mechanical properties of MKPC for radioactive waste encapsulation, there is no specific legislation to date. Nonetheless, according to the United Kingdom Nuclear Decommissioning Authority (UK NDA), the waste containers for the existing designs of unshielded waste packages must provide enough mechanical strength to withstand stacking forces, resist damage due to pressurization by internally generated gases, ensure waste package performance under accident conditions and withstand other loads that may occur during their long-term management [32]. The UK NDA suggests that a compressive strength between 4 MPa and 40 MPa should be sufficient to ensure these requirements [33].

Therefore, this work aims to characterize MKPC pastes and mortars using alternative fillers, such as pumice, wollastonite, and two different types of metakaolin, designed for the encapsulation of radioactive waste. For this, different formulations were carried out and characterized by mechanical studies, such as compression strength, dimensional stability, workability, and mineralogical studies by X-ray diffraction (XRD) and Scanning Electron Microscopy—Energy Dispersive X-ray spectroscopy (SEM-EDX) at curing times of 7, 28 and 90 days, in isolated curing conditions with a fixed molar ratio of MgO/KH_2_PO_4_ = 1.

## 2. Materials and Methods

### 2.1. Raw Materials

The MgO was supplied by Martin Marietta Inc. with the commercial name of MAGCHEM 10CR. This is a hard-burnt MgO with a purity of 98 wt.%, a specific surface area of 0.98 ± 0.02 m^2^/g, and a mineralogical composition of periclase according to the XRD results. The KH_2_PO_4_ is a highly soluble salt, sold as fertilizer by YARA under the commercial name of KristaTM, with a purity of >98 wt.%. A laboratory-grade boric acid with a purity of >99.5 wt.% was used as setting retarder.

Coal fly ash (Class F—low calcium) was provided by the Cordemais thermal power station in France, operated by Électricité de France S.A, with a particle size distribution of d10 = 7 μm, d50 = 31 μm, d90 = 163 μm, and chemical composition based on aluminosilicates in the mineral form of quartz, mullite, and amorphous phases.

Wollastonite was provided by Crimidesa. It is mainly composed of CaO and SiO_2_ and the mineralogy contains, in addition to wollastonite (CaSiO_3_), quartz (SiO_2_), albite (NaAlSi_3_O_8_) and calcite (CaCO_3_), and in minor proportion K-feldspar (KAlSi_3_O_8_), muscovite (KAl_2_(AlSi_3_O_10_)(OH)_2_), diopside (MgCaSi_2_O_6_) and gypsum (CaSO_4_.H_2_O) according to the XRD analysis.

Pumice was provided by Kremer with a grain size of <40 μm. The mineralogy contains mostly silica rich glass and a minor number of silicates, such as alkali feldspar, quartz, and biotite.

Two different types of metakaolin (MK) were evaluated. One was provided by Imerys, with 95 wt.% of particles of <80 μm, named in this work as MKA, and the second one, named as MKB, was provided by IMCD, with 68 wt.% of particles of <2 μm. In general, both MK samples are quite similar in chemical composition and mineralogy, composed mainly of amorphous aluminosilicates, anatase, and quartz. The chemical composition of all the fillers measured by X-ray Fluorescence (XRF) can be observed in Table 1.

### 2.2. Preparation of MKPC Mortars and Pastes

Table 2 presents the designed mix proportions of MKPC pastes and mortars, including the filler, MgO, KH_2_PO_4_, water, H_3_BO_3_, and sand (when cement mortars were formulated). The fresh MKPC was prepared by pre-mixing the MgO, KH_2_PO_4,_ and the corresponding filler for 2 min, then the water with dissolved H_3_BO_3_ was added to the solid powder and mixed for 5 min using a mechanical stirrer. The molar ratio MgO/KH_2_PO_4_ was fixed to 1 in all the formulations to maintain the pH close to neutrality. For the measurement of mechanical properties, a first batch of MKPC mortars was prepared with a fixed filler/cement (cement = MgO + KH_2_PO_4_) mass ratio of 1.00 and an H_2_O/cement mass ratio of 0.51 for FA and WO and 0.75 for PM and 0.65 for MKA, respectively. This was the minimum content of water required to obtain adequate workability to allow the handling and pouring of the mixtures. Due to the importance of the H_2_O/cement ratio in the mechanical properties, a second batch of MKPC mortars was prepared with a lower filler/cement mass ratio of 0.40 for PM, MKA, and MKB, with a fixed H_2_O/cement mass ratio of 0.51. Finally, a sand/cement mass ratio of 1.00 was used for all mortar formulations.

For the chemical characterization of MKPC pastes, 5 formulations were prepared with a fixed H_2_O/cement mass ratio of 0.40 and a fixed filler/cement mass ratio of 1.00 for FA and WO and 0.40 for PM, MKA, and MKB. An H_3_BO_3_/cement mass ratio of 0.02 was used in all formulations. All samples were cured in sealed plastic bags at 20 ± 2 °C, within a curing chamber, for periods of 7, 28, and 90 days.

The compressive strength were measured for specimens of 40 × 40 × 160 mm^3^ after 7, 28, and 90 days of curing at a loading rate of 50 N/s with an AUTOMAX PRO compression tester according to the European norm EN 196-1:2016 [34]. The dimensional stability was also measured in specimens of 40 × 40 × 160 mm^3^ after 7, 28, and 90 days of curing using a Matest Length Comparator E077; the test consists of inserting metal pins at the two ends of the sample and measuring the deformations after the cement has been set. The measurement is made vertically using an extensometer on the set sample that has been removed from the mold. The workability was measured in mortars by means of the mini-slump test. In this test, an open cone of 57 mm height and diameters of 19 mm at the top and 38 mm at the base was filled with the sample and lifted immediately afterward. After 1 min, a digital photograph was taken perpendicularly above the resulting layer of material, from which the slump area was calculated using ImageJ software, calibrated using a scale [35]. The initial and final setting time was measured using an automatic Vicat Needle Matest Vicatronic E044N according to the ASTM–C191 norm [36]. The compressive strength, dimensional stability, and workability were measured in MKPC mortars with a sand/cement mass ratio of 1. To stop the acid-base reaction at the different times of curing, and prior to any analytical determination, all the samples were immersed in ethanol 96 wt.% and dried at 45 °C. Duplicate measurements were carried out for each MKPC formulation, obtaining the mean and standard deviation.

The chemical and mineralogical characterization was carried out in MKPC pastes to avoid the chemical interference of quartz and other minerals present in the sand. The powder crystalline phases of the pastes were identified after 7, 28, and 90 days of curing by X-ray diffractometry (XRD), measured at an angle from 3° to 70° 2θ with a Cu X-ray source (λ(Kα1) = 1.54056 Å) in a BRUKER D8 equipment. The final identification of the phases was achieved with the X’PERT Highscore Plus^®^ software using the mineralogical database Powder Diffraction File-2 (PDF-2™) [37].

The micro-morphology and chemical composition of the MKPC pastes were observed in fresh fracture samples coated in gold by SEM-EDX using a JEOL JM-6400 microscope coupled with a LINK LZ_5 EDX analyzer. The pH of the porewater solution of the MKPC pastes was measured at up to 95 days of curing. Samples were crushed and powder grinded, then pH was measured in suspensions stirred for 5 min with degasified deionized water and with a water/solid ratio of 1 [38]. Determination of the porewater for pH determination by porepressing, such as described in [39], was discarded because of the high density and low porosity of these systems.

## 3. Results and Discussion

### 3.1. Mechanical Characterization

**Figure 1:**
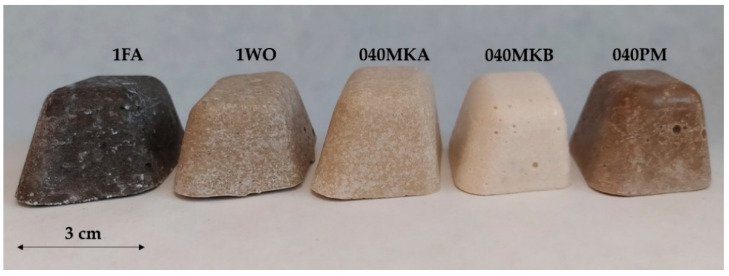
Appearances of the samples of MKPC pastes after 7 days of curing.

The appearances of the MKPC paste samples with the different fillers at water/cement mass ratios of 0.40 are displayed in Figure 1. None of the samples showed segregations or fractures, showing a good mechanical and physical constitution. Furthermore, very little to no efflorescence was observed in all formulations, suggesting the complete reaction of KH_2_PO_4_ in the sealed bag and low humidity curing conditions.

#### 3.1.1. Fresh Paste Properties: Workability and Setting Time

The results of setting time and workability of the fresh pastes are shown in Table 3. The initial setting time indicates the time to which the cement can be molded without losing consistency while the final setting time indicates the time when the cement completely loses its plasticity. In general, MKPC are known to exhibit shorter setting times than OPC, although slow setting leads to faster strength development [40]. All the formulations showed similar setting times except for the formulation 1WO, with a final setting time significantly longer than the rest of the formulations produced with an addition of filler. Additionally, a formulation without filler was also prepared. It was found that all formulations with fillers had shorter final setting times than those obtained for MKPC alone (5.8 h), likely due to an increase in the contact surface area in the presence of filler, which increases the reactivity of the cement.

The flow areas of the cement pastes, that allowed for the evaluation of the workability of the fresh mortars measured by the mini-slump tests, resulted between 35 cm^2^ and 40 cm^2^ for all the formulations. These results indicate that the workability was suitable for the manipulation of the fresh mixture and its pouring into molds. For the formulations 040PM, 040MKA and 040MKB it was only possible to incorporate 40% of filler, with respect to the cement content, due to the higher porosity and larger capacity of water retention by the fillers. At higher contents of filler, the mixtures were excessively dry and exhibited poor workability.

#### 3.1.2. Compressive Strength

**Figure 2:**
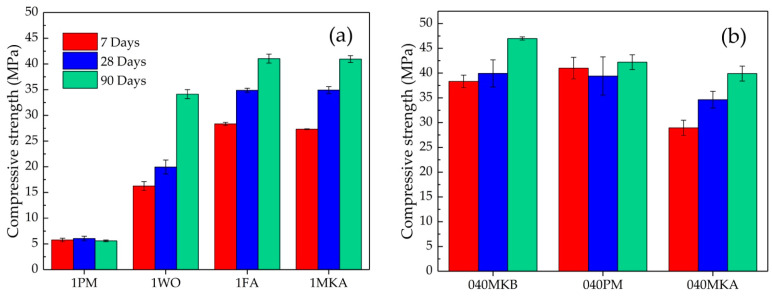
Compressive strength of MKPC mortars. (**a**) Mortars with a fixed filler/cement mass ratio of 1.00; (**b**) mortars with a fixed water/cement mass ratio of 0.51.

Figure 2a shows the compressive strength of MKPC mortars 1FA, 1WO, 1PM, and 1MKA with a fixed mass ratio filler/cement of 1.00 after 7, 28, and 90 days of curing. The formulation with PM revealed the lowest values of 5.6 MPa after 90 days of curing due to the higher amount of water (water/cement mass ratio = 0.75), being discarded due to its low strength and consistency. Similar results were reported by Ding and Li [41], showing a decrease in compressive strength with higher water/cement ratios. The authors attribute these results to the formation of a more porous structure that decreases the strength performance severely. The compressive strength for the mortar with MKA after 90 days of curing was 41 MPa, despite the high water/cement mass ratio (0.65). Nevertheless, a high amount of water may be undesirable due to the formation of hydrated intermediate phases instead of K-struvite [42], discarding this formulation from further analyses. The formulations with FA and WO showed compressive strengths of 41 and 34 MPa at 90 days, respectively. Several authors reported similar results of compressive strength in a range of 24–27 MPa, using FA as filler under curing conditions of 20 °C and >95% relative humidity after 28 days (e.g., [11,43]). Moreover, comparable results of 45 MPa were reported using wollastonite as filler, after 28 days of curing in air at 20 °C and relative humidity of 70% [23].

The results of compressive strength for the second batch of MKPC mortars 040PM, 040MKA, and 040MKB with a filler/cement mass ratio = 0.40 and water/cement mass ratio = 0.51 are shown in Figure 2b. The three formulations revealed similar compressive strengths after 90 days of curing and are comparable to those found using FA. According to literature, the improvement in the mechanical properties using MK is attributed to the formation of lower porosity microstructures and denser interfaces between the metakaolin particles and hydration products in the MKPC [26,27]. Since pumice is a novel material as filler in MKPC, it was not possible to find comparable results in the literature. However, its similarity with the other fillers used in this work may suggest good performance at lower water/cement mass ratios.

The compressive strength of all formulations increased gradually with curing time, with a 52% higher strength for the 1WO formulation after 90 days of curing with respect to 7 days, and 31%, 28%, 18%, and 1.7% for the 1FA, 040MKA, 040MKB, and 040PM formulations, respectively. These increases in compressive strength with time indicate the continuity of the K-struvite formation reaction and a more condensed structure, resulting in an improvement in the mechanical properties of the MKPC. All formulations presented in this work fulfill the compressive strength recommended by the UK NDA, necessary to ensure the performance of the waste packages [33], although the 1PM mortar presented a low value.

#### 3.1.3. Dimensional Stability

**Figure 3:**
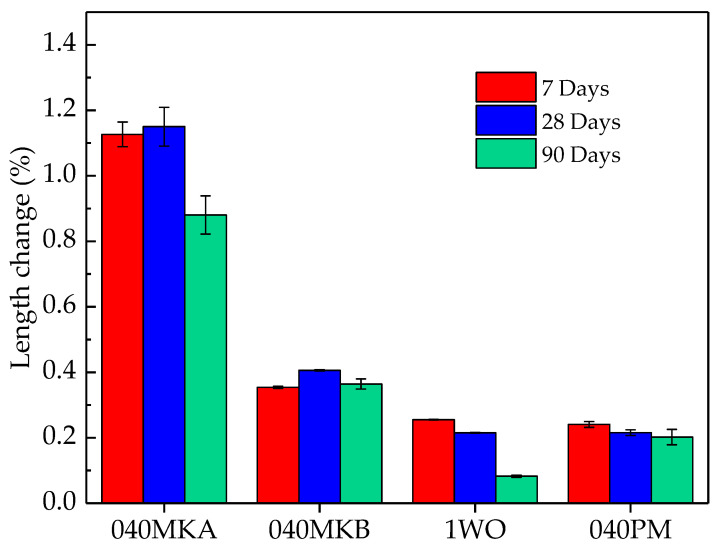
Length expansion of MKPC mortars with a mass ratio H_2_O/cement = 0.51 and a mass ratio filler/cement = 1 for WO and 0.40 for PM, MKA and MKB.

The length change of mortars with a water/cement mass ratio = 0.51 and a filler/cement mass ratio = 1 for 1WO and 0.40 for 040PM, 040MKA, and 040MKB was calculated by measuring the length change after 7, 28, and 90 days of isolated curing at 20 °C, as shown in Figure 3. Previous works have demonstrated that the Mg/PO_4_ molar ratio and the degree of humidity of the curing conditions are the main factors in the length change of MKPC, with the expected low impact of the filler in the MKPC system [42]. For the FA formulation, it was not possible to measure the dimensional stability due to the limited availability of this material in our study; nonetheless, results obtained by De Campos et al. [44] for a similar formulation with the same water/cement and filler/cement ratios and curing conditions used in this work show a percentage of expansion of 0.1% after 28 days of curing.

The major relative length change (%) is measured for the formulation 040MKA with a percentage of expansion of 1.1% after 28 days of curing, followed by a shrinkage to 0.9% after 90 days. Similar results were found for the 040MKB sample with a percentage of expansion below 0.4% after 90 days of curing. For the fillers 1WO and 040PM, the increase in expansion from 7 to 28 days is not observed. A continuous decrease is observed from the original length as a function of time, although these length changes are very low, indicating excellent dimensional stability. The results obtained in this work on dimensional stability are difficult to compare with the available literature due to the varying types of curing, raw materials, and measurement conditions in all other experimental studies. Nonetheless, several works evaluate the dimensional change of MKPC in absence of a filler, allowing this to compare the impact of the filler in the MKPC system. Le Rouzic et al. [24] and Li and Chen [45] evaluated the dimensional swelling of MKPC without filler materials, finding a length change after 7 days of curing of 0.2% and 0.1%, respectively, meaning these results are in the same magnitude order as those found in the present work, with the exception of the 040MKA sample, suggesting a low influence of the filler materials in the dimensional change development of the MKPC. Besides the relative swelling found in all formulations, no cracking or damage was observed in any of the samples evaluated in the present study.

### 3.2. Chemical Characterization

#### 3.2.1. pH Measurement

**Figure 4:**
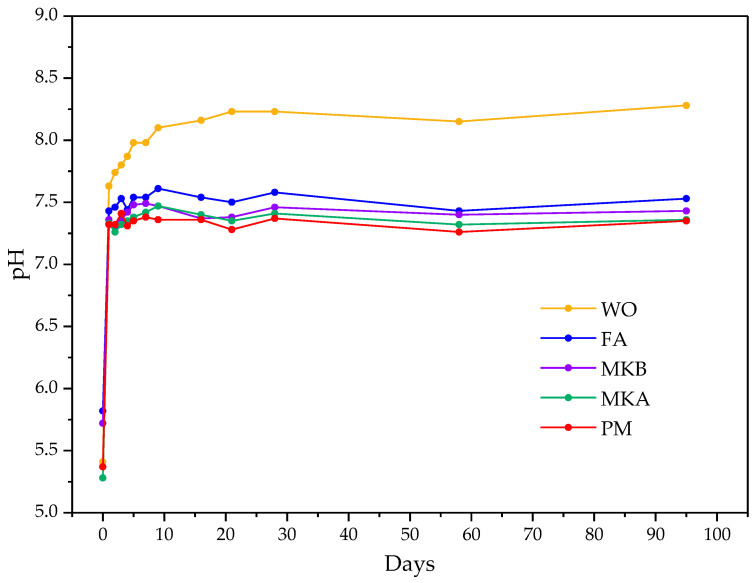
pH of MKPC paste suspensions with a solid/liquid ratio of 1:1.

Figure 4 shows the results of pH evolution in the suspensions of MKPC pastes with a 1:1 solid/liquid ratio as a function of the curing time, up to 90 days [38]. The pH is an important parameter for the encapsulation of reactive metals due to its influence on corrosion processes. At the initial time (t = 0), all formulations showed a pH in the range of 5–6, controlled mainly by the KH_2_PO_4_ and H_3_BO_3_ dissolved in the fresh MKPC paste. The formulations with the fillers FA, PM, MKA, and MKB show very similar pH values between a range of 7 and 7.6 for the first 90 days of curing, corresponding this pH range to the corrosion passivation zone of aluminum; therefore, no corrosion and lower risk for hydrogen evolution will occur at this pH range [15]. The higher pH was measured for the formulation with WO due to the possible partial dissolution of the wollastonite, adding Ca and generating a more alkaline medium (see Section 3.2.2) that could contribute to increase the pH to the actual range of 8–8.5. Nonetheless, pH under 9 is still adequate for the encapsulation of reactive metals, and all the fillers of this work are suitable for this type of application in terms of alkalinity [46].

The MgO/PO_4_ is one of the main factors affecting the pH in MKPC. Although the theoretical molar ratio MgO/PO_4_ value for a complete MgO reaction is 1, according to the reaction Equation (1), more magnesia is needed in a mix to guarantee strength development [47]. Nonetheless, in the case of radioactive waste encapsulation, the strength development is not the main factor to consider (a minimum of 4 Mpa according to the UK NDA); what is desirable is the minimum amount of MgO to obtain a neutral pH to reduce the risk for hydrogen evolution processes.

#### 3.2.2. Mineralogical and Microstructural Analysis (XRD and SEM-EDX)

**Figure 5:**
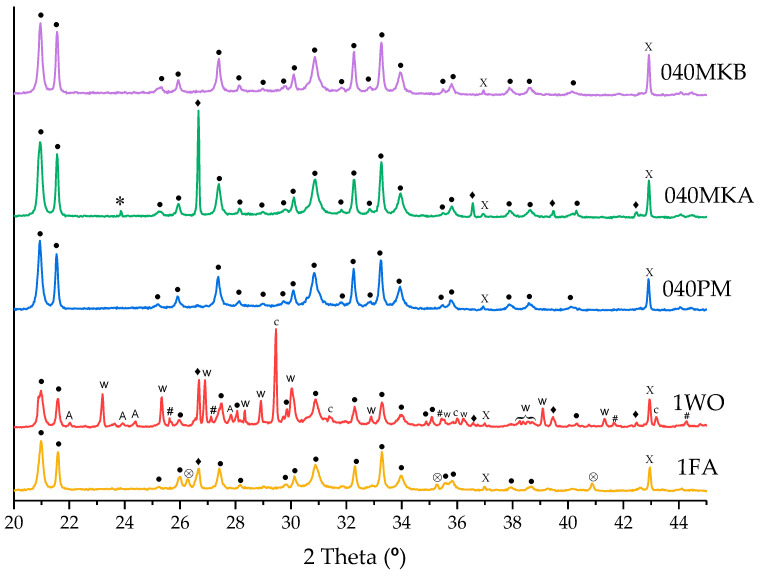
XRD patterns of MKPC pastes after 28 days of curing. ●: K-struvite (PDF file: 01-075-1076); X: MgO (PDF file: 01-087-0651); W: wollastonite (PDF file: 01-075-1396); ♦: quartz (PDF file: 01-079-1910); *: KH_2_PO_4_ (PDF file: 00-035-0807); ⨂: mullite (PDF file: 01-083-1881); c: calcite (PDF file: 01-083-0578); #: microcline (PDF file: 00-022-0675); A: albite (PDF file: 01-076-0927).

The XRD patterns of the hydrated MKPC cement paste after 28 days of curing are shown in Figure 5. The main hydration product for all formulations is observed to be crystalline K-struvite (sharp XRD reflections). A small reflection of unreacted MgO is also observed in all formulations; nonetheless, presence of KH_2_PO_4_ is not observed, except for the formulation 040MKA with a small reflection at 23.8° 2θ after 28 days, which is no longer present after 90 days of curing. Therefore, either the complete reaction of KH_2_PO_4,_ or a deficient grind of the paste prepared for its study after 28 days of curing occur. A decrease in the reflections attributed to MgO was also observed after 90 days, suggesting the continuity of the hydration reaction. Furthermore, no evidence of the formation of hydrate intermediates, such as Mg_2_KH(PO_4_)_2_·15H_2_O, was found [48], although a different type of XRD study should have been performed to study short-term intermediates (e.g., time-resolved synchrotron XRD [12]).

The addition of the different fillers in the MKPC does not seem to form new crystalline phases, besides those already found in the filler materials. The main hydration products are almost identical in all formulations, indicating a negligible chemical behavior of all the fillers studied in the MKPC matrix.

**Figure 6:**
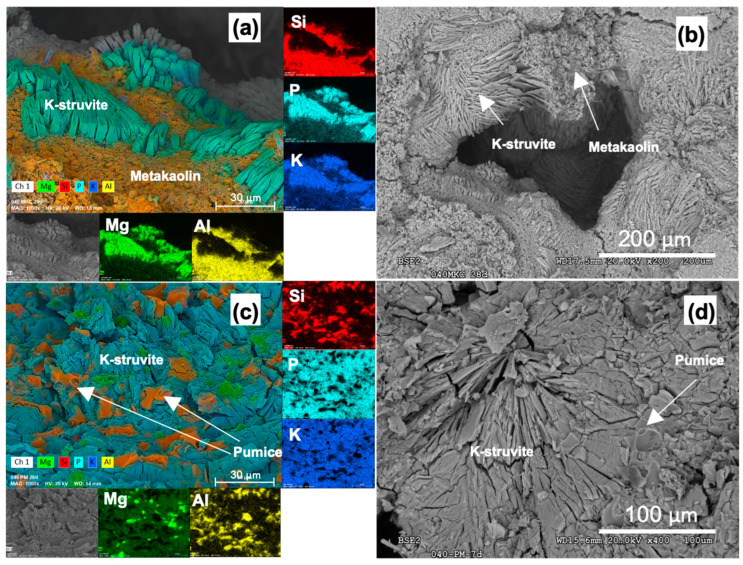
SEM images of MKPC pastes with MKB (**a**,**b**) and PM (**c**,**d**) fillers in fresh fractures.

**Figure 7:**
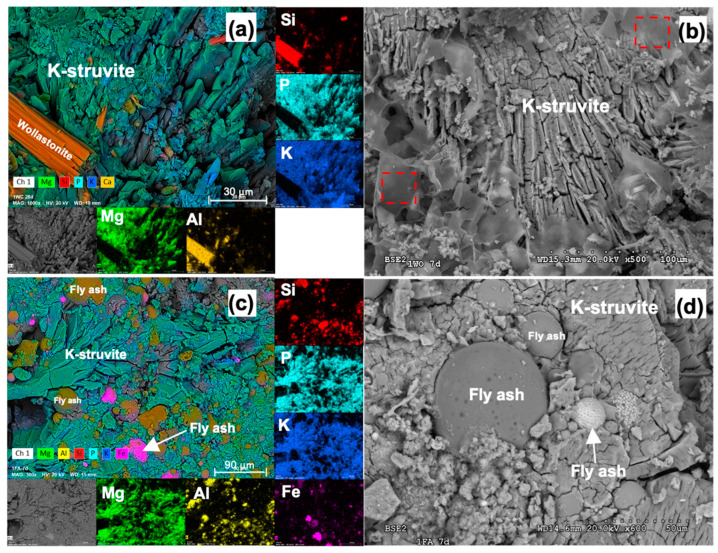
SEM images of MKPC pastes with WO (**a**,**b**) and FA (**c**,**d**) fillers in fresh fractures.

Image analyses were performed using SEM to study the microstructure of the different MKPC formulations. Figure 6 and Figure 7 show SEM images and EDX elemental distribution maps for MKPC paste formulations. Crystals of K-struvite can be observed in all formulations, surrounded by filler materials. According to Mo et al. [27], K-struvite can develop various morphologies depending on the location where it formed. In the pores, K-struvite tends to grow into large tabular crystals (as observed in Figure 6a,b). In confined regions K-struvite tends to adopt less regular but denser aggregated crystals (as observed in Figure 6c,d). Punctual EDX analyses on K-struvite crystals present small amounts of Si and Al (<1%), denoting a low incorporation of these elements in the MKPC matrix. These concentrations are too low to be observed in the EDX elemental distribution maps, where Mg, K and P are dominant, and the Mg/P and K/P atomic ratios remain close to 1 according to the K-struvite theoretical stoichiometry. These results are consistent with those found by Gardner et al. [43], where Si and Al were incorporated in the MKPC matrix blended with FA. This was attributed to the partial dissolution of the aluminosilicate glassy fraction in the FA, leading to the formation of potassium aluminosilicate phosphate hydrates, and due to the chemical similarity of FA with the rest of the fillers used in this work, a similar process is likely to occur.

For the formulations with MKB (Figure 6a,b) the morphology presented well developed crystal aggregates with networks of layered K-struvite crystals surrounded by amorphous metakaolin fulfilling the space between layers. This could explain the higher compressive strength of the MKPC blended with this filler by developing a denser microstructure, filling the cracks and generating a less porous structure. Additionally, the development of crystal growth of the K-struvite crystals layered in random orientation could lead to a higher mechanical strength due to the absence of potential weakened planes in the structure of the MKPC. Particles of massive aluminosilicate aggregates (no crystalline habit evidence) attributed to pumice can be observed embedded in the MKPC matrix (Figure 6d). Green spots of unreacted MgO can also be observed in Figure 6c, indicating its incomplete reaction, in agreement with the results obtained by the XRD analysis. Nonetheless, in both cases, a good bonding between the filler and the MKPC matrix and absence of new mineral phases was observed.

Similar structures were found for the MKPC formulations with WO and FA fillers as shown in Figure 7. In the pastes prepared with WO (Figure 7a), rod shape crystals of wollastonite can be observed embedded in the MKPC matrix. Glassy morphologies were also found in this sample, as shown in Figure 7b with dashed line red square markers. Punctual EDX semiquantitative analyses of these areas were performed, finding relatively high contents of Ca (between 3 and 10%) and S (between 1.8 and 13.7%), possibly attributed to secondary minerals in the filler WO. Nonetheless, despite the minor contents of Ca, Si, and Al detected in the K-struvite crystals with the different types of fillers studied in this work, no new mineral phases were found by the XRD analyses, indicating the amorphous character of this phase and its negligible impact.

**Figure 8:**
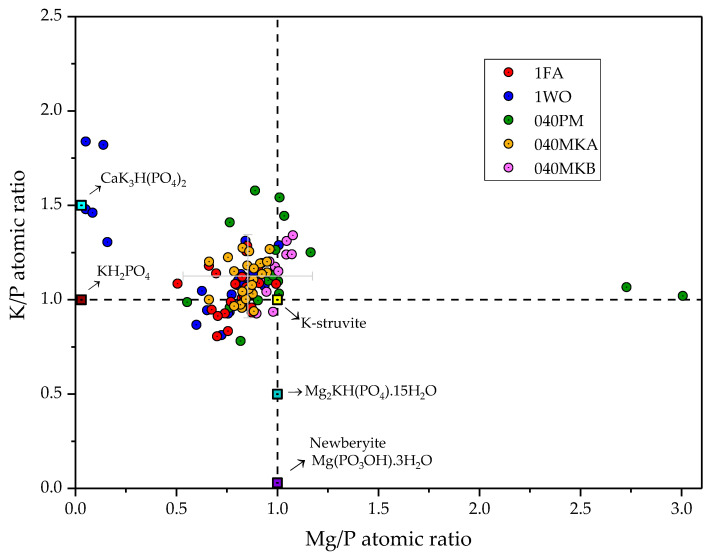
Atomic ratios of K/P and Mg/P in MKPC pastes after 28 days of curing measured by semiquantitative EDX analyses.

In order to further understand the chemistry of the system, multiple punctual EDX semiquantitative analyses were performed in MKPC pastes with all fillers after 28 days of curing (Figure 8). The results are expressed in Mg/P and K/P atomic ratios to compare them to the theoretical stoichiometric ratios of potential mineral phases that could form as hydration intermediates in the K-struvite formation system under the experimental conditions [23]: brushite (CaKHPO_4_·2H_2_O), newberyite [Mg(PO_3_OH)·3H_2_O], CaK_3_H(PO_4_)_2_ and Mg_2_KH(PO_4_)_2_·15H_2_O. The averaged K/P and Mg/P atomic ratios fall within the range of 1.1 ± 0.2 and 0.9 ± 0.3, respectively, indicating a composition close to stoichiometric K-struvite, with a lower content in Mg, probably due to the low Mg/P molar ratio of 1 used in all formulations and the incomplete reaction of MgO. A large dispersion in the Mg/P ratio is observed for the formulation 040PM, with two points offset with an Mg/P ratio of 2.7 and 3.0. These last results are in agreement with the heterogeneous dispersion of unreacted MgO, observed by the SEM-EDX analyses, that also confirm the absence of reaction intermediates with different K/P and Mg/P ratios, and is in agreement with the observations by XRD (Figure 5).

## 4. Conclusions

All the filler materials used in the present study (fly ash, wollastonite, metakaolin and pumice) were incorporated in the MKPC formulations, obtaining samples without efflorescence and with good consistency in isolated curing conditions. However, the fillers metakaolin and pumice were required to be added in a lower filler/cement ratio (40%) in order to maintain a water/cement ratio equal to 0.51 in mortars and 0.40 in pastes.

All mortars prepared in this work presented high compressive strengths for the encapsulation of metallic radioactive waste. Additionally, the percentage of dimensional expansion for the fillers pumice, wollastonite and metakaolin B is comparable to results presented in the literature for MKPC without filler, suggesting a low impact of this fillers in MKPC systems.

The pH of the cement paste suspensions in a water/solid ratio of 1 was found to be less than 8.5, corresponding to the passivation zone of aluminum; therefore, no corrosion should be expected under these encapsulation conditions. No hydration intermediates of K-struvite or new mineral phases were found after 28 days, other than those previously found in the filler materials. Unreacted MgO was observed, with the intensity of its main XRD reflection decreasing with time, indicating the continuity of the K-struvite formation reaction.

No new mineral phases could be identified by SEM. EDX analyses confirmed averaged atomic ratios K/P and Mg/P of 1.1 ± 0.2 and 0.9 ± 0.3, respectively, corresponding to the theoretical stoichiometric ratio of K-struvite. The lower Mg/P than K/P ratio was attributed to the incomplete reaction of MgO.

All the filler materials presented in this work showed favorable results for their use in MKPC cements for the encapsulation of radioactive metal waste. The wollastonite filler, being a natural material of easy access–low cost and being able to be incorporated in a higher proportion in the MKPC mixture, is particularly attractive. However, further study of the MKPC–metal interaction is needed in order to further understand the corrosion processes within the MKPC systems.

## Figures and Tables

**Table 1:** Chemical composition and specific surface area of filler materials.

wt.%	Fly Ash	Wollastonite	Pumice	Metakaolin A	Metakaolin B
SiO_2_	52.9	41.21	55.4	57.31	52.94
Al_2_O_3_	21.85	4.98	17.65	37.01	43.18
CaO	3.79	43.31	1.23	0.11	0.02
Fe_2_O_3_	10.55	3.68	3.26	1.62	0.54
MgO	1.55	2.45	-	0.32	-
K_2_O	1.77	1.64	9.7	0.73	0.19
Na_2_O	1.24	1.26	10.24	-	0.31
TiO_2_	0.97	0.54	0.32	2.37	2.47
P_2_O_5_	0.15	-	0.04	-	0.11
$F^{-}$	-	-	0.53	-	-
$Cl^{-}$	-	-	0.36	-	-
∑ others	0.81	0.94	1.29	0.54	0.25
SSA* (m^2^/g)	11.68	6.54	2.69	21.28	12.61

SSA* Specific surface area measured by BET method of nitrogen adsorption.

**Table 2:** Formulations of MKPC pastes and mortars.

Filler	MgO/KH_2_PO_4_(Molar)	H_2_O/Cement (Mass)	Filler/Cement (Mass)	Sand/Cement (Mass)	H_3_BO_3_/Cement (Mass)
Mortars					
1FA	1.00	0.51	1.00	1.00	0.02
1WO		0.51	1.00		
1PM		0.75	1.00		
040PM		0.51	0.40		
1MKA		0.65	1.00		
040MKA		0.51	0.40		
040MKB		0.51	0.40		
Pastes					
FA	1.00	0.4	1.00	0.00	0.02
WO			1.00		
PM			0.40		
MKA			0.40		
MKB			0.40		

Cement: MgO + KH_2_PO_4_. FA: fly ash; WO: wollastonite; PM: pumice; MKA: metakaolin A; MKB: metakaolin B.

**Table 3:** Setting time and workability results of the MKPC mortars.

Formulation	Initial Setting Time (h)	Final Setting Time (h)	Flow Area (cm^2^)
1FA	2.0	4.0	34.3 ± 4.5
1WO	3.2	6.2	40.9 ± 2.4
040PM	2.2	2.6	39.0 ± 3.2
040MKA	2.0	2.4	41.9 ± 1.6
040MKB	1.7	2.1	36.9 ± 1.1
No Filler	5.2	5.8	81.3 ± 0.7

## Data Availability

Not applicable.
